# The evolution and maintenance of virulence in microparasites.

**DOI:** 10.3201/eid0202.960203

**Published:** 1996

**Authors:** B R Levin

**Affiliations:** Emory University, Atlanta, Georgia 30322, USA. biobrl@emuvm1.cc.emory.edu

## Abstract

In recent years, population and evolutionary biologists have questioned the traditional view that parasite-mediated morbidity and mortality¿virulence¿is a primitive character and an artifact of recent associations between parasites and their hosts. A number of hypotheses have been proposed that favor virulence and suggest that it will be maintained by natural selection. According to some of these hypotheses, the pathogenicity of HIV, Vibrio cholerae, Mycobacterium tuberculosis,theShigella,as well as Plasmodium falciparum,and many other microparasites, are not only maintained by natural selection, but their virulence increases or decreases as an evolutionary response to changes in environmental conditions or the density and/or behavior of the human population. Other hypotheses propose that the virulence of microparasites is not directly favored by natural selection; rather, microparasite-mediated morbidity and mortality are either coincidental to parasite-expressed characters (virulence determinants that evolved for other functions) or the product of short-sighted evolution in infected hosts. These hypotheses for the evolution and maintenance of microparasite virulence are critically reviewed, and suggestions are made for testing them experimentally.


### The Evolution and Maintenance of Virulence in Microparasites

**Bruce R. Levin, Ph.D.**
Emory University, Atlanta, Georgia, USA

In recent years, population and evolutionary biologists have questioned the traditional view that parasite-mediated morbidity and mortality—virulence—is a primitive character and an artifact of recent associations between parasites and their hosts. A number of hypotheses have been proposed that favor virulence and suggest that it will be maintained by natural selection. According to some of these hypotheses, the pathogenicity of HIV, *Vibrio cholerae*, *Mycobacterium tuberculosis*, the *Shigella*, as well as *Plasmodium falciparum*, and many other microparasites, are not only maintained by natural selection, but their virulence increases or decreases as an evolutionary response to changes in environmental conditions or the density and/or behavior of the human population. Other hypotheses propose that the virulence of microparasites is not directly favored by natural selection; rather, microparasite-mediated morbidity and mortality are either coincidental to parasite-expressed characters (virulence determinants that evolved for other functions) or the product of short-sighted evolution in infected hosts. These hypotheses for the evolution and maintenance of microparasite virulence are critically reviewed, and suggestions are made for testing them experimentally.

How much of the emergence and reemergence of infectious diseases is due to evolution, rather than ecological, technical, and social change (1)? Under what conditions will attenuated vaccine organisms become virulent? Are hospitalized and immunocompromised hosts reservoirs for the evolution of virulent pathogens (2)? The answers to these and related questions require an understanding of the ecological conditions and genetic processes responsible for the evolution and maintenance of parasite-mediated morbidity and mortality in infected hosts—virulence, as we shall define it here.

At least since Darwin's time (3), evolutionary biologists have been interested in infectious diseases, but primarily with respect to the role of these diseases in the adaptation and evolution of humans and other species (4). A bit more than 15 years ago, this interest in infectious disease took a new turn, a focus on the microbes responsible for these diseases and the evolution and maintenance of their virulence. Here I offer a relatively brief and personal review of current theories of the evolution and maintenance of virulence in the bacteria, viruses, protozoa, and single cell fungi, "microparasites" (to use the term employed by population biologists), responsible for infectious diseases. I consider how these theories fit, what is known about the epidemiology of microparasite infections and the mechanisms of pathogenesis, and discuss procedures to test hypotheses derived from these theoretical considerations of the population biology and evolution of microparasites. For other recent reviews of this subject, see (5-8).

Address for correspondence: Bruce R. Levin, Ph.D., Emory University, EcLF, 1510 Clifton Road, N.E., Atlanta, GA 30322, USA; fax: 404-727-2880; e-mail: biobrl@emuvm1.cc.emory.edu.

### The Conventional Wisdom

At one time, virulence was almost universally considered an artifact of recent associations between parasites and their hosts (9, 10), and to a fair extent, it still is (11). In accord with this view, which Bob May and Roy Anderson called "conventional wisdom" (12), parasite-host coevolution is necessarily in the direction of commensalism or, nicer yet, mutualism. The logic behind this view is pleasing to human sensibilities. A fully evolved parasite would not harm the host it needs for its survival, proliferation, and transmission. Indeed, the appeal of this view of nature of parasite-host coevolution was sufficient for its corollary to also be assumed valid. That is, pathogenesis is often taken as evidence of recent associations between parasites and their hosts.

Many observations are consistent with conventional wisdom about parasite-host coevolution. This is particularly so for most of the so-called emerging diseases. For example, Legionnaires' disease, Lyme disease, and pneumonia caused by hantavirus are consequences of human infection by parasites and/or commensals of other species, rather than by organisms that have had a long association with humans. In fact, for these emerging diseases and some older microparasitic diseases, like Rocky Mountain spotted fever, anthrax, and rabies, humans play no (or at best a negligible) role in the transmission of the parasite and, in that sense, are an evolutionary dead end. While HIV is transmitted between humans, its association with our species is almost universally considered recent (13, 14).

Other observations can be interpreted as inconsistent with conventional wisdom. For some virulent pathogens, like *Shigella* and *Neisseria gonorrhoeae*, humans appear to be either the unique or the dominant host and vector for infectious transmission (15). For other lethal microparasitic diseases like malaria and tuberculosis (TB), there is evidence that these microparasites have had a long history in human populations and that humans play a major if not unique role in their infectious transmission. However, for the pathogens involved in both these diseases, animal origins have been implicated, and it is difficult to find clear evidence of their existence (or that of other extant pathogens) before the origins of agriculture (16-18).[1] One can always rescue conventional wisdom from these inconsistent observations by assuming that "long" is not long enough for these microparasites to evolve or coevolve with humans to a more amenable relationship. Then again, it may well be that some microparasites responsible for new infections in human hosts will evolve to become increasingly virulent human pathogens and be readily transmitted between human hosts.

Conventional wisdom is not based on hypotheses that can be readily tested and rejected. Microparasites that lead to the extinction of their only host face the same fate as the host. On the other hand, evolving and becoming gentle and prudent in treating their hosts (when natural selection operating at the level of individual microparasites favors profligate behavior like virulence) require some form of group-level or kin selection (8), and/or a host evolutionary response that unilaterally converts an otherwise virulent microparasite into a commensal. Conventional wisdom does not account for the actual mechanisms responsible for the evolution of benign associations between microparasites and their hosts.

### Epidemiologic Models and the "Enlightenment"

In the early 1980s, at least among evolutionary biologists, conventional wisdom gave way to what, in an earlier consideration of this subject Catharina Svanborg and I satirically (but sympathetically) referred to as the "enlightenment" (24). In accord with this new view, natural selection could favor the evolution and maintenance of virulence as well as commensal and symbiotic associations between microparasites and their hosts. In other words, virulence could be the evolved as well as the primitive stage of these associations. The direction of natural selection in any given situation depends on the epidemiology and ecology of the microparasite and, in particular, the relationship between its virulence and its rate of infectious transmission in the host population.[2] This can be seen in the equation for the finite rate of increase of a directly transmitted microparasite in a wholly susceptible host population (12, 25, 26)

$$R_0 = \frac{ßN}{\alpha + b + \nu}$$

[1] The existence of genetic polymorphisms, like sickle cell, thalassemia, and glucose 6-phosphate dehydrogenase (G6PH) deficiency (19, 20), Duffy-negative blood groups (21), and specific HLA alleles (22) maintained by *Plasmodium*-mediated selection can also be interpreted as evidence for malaria's long association with humans. There is evidence for inherited resistance to TB among mammals (23), and arguments that TB epidemics have selected for inherited resistance in humans (16). However, that evidence is not as compelling as that for malaria.

[2] In at least the mathematical theory, the morbidity component of microparasite virulence is not treated explicitly (25). The symptoms and pain resulting from infection are implicitly incorporated in the rates of disease-associated mortality, recovery, and transmission. Moreover, while acknowledging the existence of microparasite-mediated selection and evolution in the host population, for the most part, the enlightened view of microparasite-host coevolution has concentrated on the changes in the microparasite population. The idea is that because of the relatively longer generation times, the rate of evolution in the host population is going to be low.

where ß is the rate constant of infectious transfer of the microparasite, N the density of the susceptible host population, α the rate of microparasite-induced mortality (virulence), b the rate of microparasite-independent mortality, and ν the rate of recovery. $R_0$ is the number of secondary infections caused by a single primary infection and serves as a measure of the fitness (here and elsewhere in a Darwinian sense) of the parasite in this naive host population. At any given host density, N, this measure of fitness of the parasite is directly proportional to its transmissibility, ß, and the term of its persistence in an infected host, the reciprocal of $\alpha + b + \nu$.

If the parameters of the $R_0$ equation were independent of each other, the predictions derived from this equation would be consistent with conventional wisdom: benign parasites would evolve. That is, natural selection would favor highly transmissible ($b \rightarrow \infty$), incurable ($\nu \rightarrow 0$), commensals ($\alpha \rightarrow 0$), or symbionts ($\alpha \rightarrow -\infty$). On the other hand, if transmission and virulence, the parameters ß and α in the $R_0$ equation, were positively coupled, natural selection could favor the evolution and maintenance of some level of virulence, $\alpha \rightarrow 0$, in the microparasite population.

In accord with the epidemiologic perspective implicit in the $R_0$ equation, an understanding of the evolution of virulence in microparasites comes down to elucidating the relationship between the rate at which the microparasite is transmitted between hosts and the rate of parasite-mediated mortality in individual infected hosts. If that relationship is positive, then some level of virulence may be favored. And, since the first statements of this new view of parasite-host coevolution (12, 26, 27), much of the research on the evolution of virulence has focused on the association between these two components of parasite fitness.

The most cited, and to me the single most compelling, evidence in support of this new interpretation of microparasite-host coevolution comes from the "experiments" using myxoma virus to control European rabbit populations in Australia and Europe (26, 28, 29). Within a relatively short time after the release of highly virulent myxoma, the viruses recovered from the then decimated and sometimes more resistant wild rabbit populations were less virulent and had lower rates of disease-induced mortality on control laboratory rabbits than those initially released. However, the extent to which myxoma virus from the wild became attenuated was substantially less than that which could be achieved experimentally (29). This was interpreted as evidence for a positive coupling between the rates of infectious transmission and rates of virus-induced mortality, a trade-off between virulence and transmission. Highly virulent forms of the virus had a disadvantage because they killed the rabbits too quickly and thus reduced the time available for them to be picked up by the insect (mosquito or flea) vectors required for their infectious transmission. Viruses that were too attenuated had a disadvantage because they generated fewer skin lesions and had lower densities of circulating virions, which presumably would reduce the rate at which they would be bitten by these insect vectors, the likelihood of biting vectors picking up myxoma, and the number of virions picked up at any given bite. Thus, in contrast to conventional wisdom and in accord with the enlightened interpretation, natural selection could favor and maintain the virulence of microparasites. This results when there is a positive coupling between a parasite's virulence and its capacity for infectious transmission.

The myxoma story is particularly compelling because the quantitative relationship between virulence and transmissibility inferred from the epidemiologic data and models was independently tested and demonstrated experimentally (30, 31). The myxoma story remains the only one for the microparasites of eukaryotic hosts where the predictions about transmission and virulence made from an interpretation of epidemiologic observations were tested experimentally. With few exceptions (32), inferences about the relationship between transmission and virulence and the trade-offs between these two attributes of a microparasite's association with its host have been derived from comparative evolution studies or retrospective interpretations of epidemiologic data. In some cases, these inferences are reasonably strong, e.g., in the study by Alan Herre (33) on fig wasps and a nematode parasite and by Deiter Ebert (34) on a planktonic crustacean with a protozoan parasite. The latter study is particularly convincing because it includes independent, experimental evidence of a positive correlation between the density of spores in infected hosts and the virulence and transmissibility of this protozoan parasite.

The enlightened view on the virulence of microparasites sometimes takes the positive association between the virulence of a microparasite and its transmissibility as axiomatic; therefore, it assumes that a microparasite's virulence is constrained solely by the need to keep the host alive to facilitate its transmission to new hosts.[3] This is implicit in much of Paul Ewald's writing on this subject (6, 35) and is the basis of his main thesis that changes in rates of infectious transmission will select for microparasite strains or species with different levels of virulence.

By assuming a necessarily positive relationship between a microparasite's capacity for infectious transmission and the extent of morbidity and rate of mortality it causes in infected hosts, a positive "trade-off" (relationship) between transmissibility and virulence, Paul Ewald has generated scenarios for the evolution of virulence and changes in virulence for a diverse array of microparasites, including those responsible for cholera, influenza, dysentery, and AIDS (6, 35). While the details of Ewald's stories may differ, the plot is almost always the same: increases in the rates of transmission favor increases in virulence, and the reverse. For example, Ewald has postulated that the virulence of HIV observed in contemporary human populations, AIDS, is in large part due to evolution in this retrovirus responding to the increases in human-human transmission rate resulting from more promiscuous sexual behavior.[4] However, even when a direct relationship between the virulence and transmission rate of HIV is assumed, a deeper consideration of the epidemiology and course of this sexually transmitted disease shows that this simplistic conclusion about evolution and the virulence of HIV is chock full of caveats (36, 37). The relative contributions of transmission and virulence (as measured by the time before the onset of AIDS) to the fitness of HIV in the population of hosts depends on whether the disease is in an epidemic or endemic phase. Moreover, as I consider later, there are other, very different, hypotheses for the evolution of the virulence of this retrovirus and other pathogenic microbes that do not require the necessarily positive association between infectious transmission and virulence upon which Ewald has based his arguments for the evolution and maintenance of virulence in microparasites.

A corollary of the hypothesis of a positive trade-off between transmissibility and virulence is that if all else were equal, increases in the degree of vertical (e.g., from a mother to a fetus) transmission of a parasite, relative to its horizontal (infectious) transmission would favor reductions in its virulence (38). There is compelling, experimental evidence to support this corollary. However, the evidence is restricted to experiments with *E. coli* and its phage, *f1*, which can be transmitted vertically, in the course of cell division, or horizontally, by infecting susceptible, uninfected bacteria (39). While some, like me most of the time, may believe in the adage "what is true for *E. coli* is true for elephants, but only more so," other, less *coli*-centric souls, may want to see more experiments of this type with microparasites and vertebrate hosts. I certainly do.

### Within-Host Population Dynamics and Virulence of Microparasites

There is a dearth of experimental investigations of the quantitative relationship between the transmission and virulence of microparasites. During the past few years, however, there has been a flurry of theoretical studies of the within-host population dynamics of microparasites that have specifically considered the relationship between the virulence and transmission rates of microparasites and their densities and/or rates of replication in infected hosts (40-44). In the simplest models developed in these theoretical studies of the within-host population dynamics of

---

[3] One way to experimentally augment the virulence of a microparasite, as, for example, measured by declines in its $LD_{50}$, is to artificially pass that microbe between hosts (15). From one perspective, this result is consistent with the trade-off hypothesis, as the effect of passage is to make the parasite's transmission independent of the host's survival, thereby allowing it to become more virulent without compromising its need to be transmitted to other hosts. However, increased virulence in a passage experiment is not sufficient evidence for that trade-off. (It may well be that the parasite's capacity for infectious transmission is impaired as a consequence of whatever increased its capacity for infectious transmission.) I know of no experiments that demonstrate that the increase in virulence generated during a passage experiment is also reflected as increased—transmissibility, as is necessary for the trade-off interpretation. Indeed, it may well be that an increase in the case-mortality rate or a reduction in the $LD_{50}$ of a microparasite will be reflected as a reduction in its natural transmissibility.

[4] I quote: "Severe immunodeficiency could develop in an old association [between a sexually transmitted virus or SIV and its host] as a result of increases in sexual partner rates causing evolution of increased virulence" (p. 143, reference 6). "If rates of unprotected sexual contact decline, so should the virulence of HIV" (p. 144, reference 6).

microparasites, the virulence of the microparasite, as measured by either the rate at which it kills its host or its $LD_{50}$, is assumed to be directly proportional to its rate of proliferation in that host, and its rate of infectious transmission is directly proportional to its within-host density (41). Under these conditions, in the absence of superinfection or mutation, selection favors microparasites with intermediate rates of within-host replication, i.e., intermediate levels of virulence. More complex situations, like the coexistence of microparasite lineages with different levels of virulence, result when virulence is proportional to the within-host growth rate of the parasite and single hosts can be infected with parasites of different growth rates (43) or when there are high rates of mutation to different levels of virulence within a host (45). Moreover, with superinfection and mutation, the theory developed in these two reports predicts that the average level of virulence of a parasite in an infected host can exceed that anticipated from models that do not allow for superinfection and/or assume that the parasite's level of virulence in an infected host remains invariant.

### The Convergence of Theories

The predictions that can be made on the basis of the current view of the evolution of virulence differ from predictions that might follow conventional wisdom because the new view allows for natural selection in the parasite population to favor the evolution and maintenance of some level of virulence. Moreover, even when there is a positive association between a parasite's virulence and its transmissibility, under the conditions described in the following paragraph, the predictions of new methods can still converge with those of conventional wisdom.

If the density of the sensitive host population is regulated by the parasite, an extension of the enlightened theory predicts that natural selection in the microparasite population can lead to continuous declines in the level of virulence, possibly to immeasurable values (46). Although not stated in this general way, the same conclusion about declining virulence can be drawn from models of the epidemiology of HIV/AIDS (36, 37). During the epidemic phase of a microparasitic infection, when the host population is composed primarily of susceptible hosts, selection favors parasites with high transmission rates and thus high virulence. As the epidemic spreads, the proportion of infected and immune hosts increases and the density of susceptible hosts declines. As a result, the capacity for infectious transmission becomes progressively less important to the parasite's Darwinian fitness and persistence in the host population. Selection now favors less virulent parasites that take longer to kill their host and, for that reason, are maintained in the host population for more extensive periods. Analogous arguments have been made for the latent period of a bacteriophage infection (47), the evolution of lysogeny (48), the tradeoff between vertical and horizontal transmission (49, 50), and the advantages of microparasite latency in general (40).

### Alternative Models for the Evolution of Microparasite Virulence

For any microparasite, the rate of transmission between hosts will always be a significant component of fitness, and, if all else is equal, parasites transmitted at higher rates in the host population have a selective advantage over less transmissible forms. On the other hand, there is no reason to assume that in general a microparasite's rate of infectious transmission will be positively associated with its virulence. Moreover, even when there is no relationship or a negative relationship between transmission and virulence, there are at least two ways by which natural selection can lead to the evolution and maintenance of virulence, coincidental evolution (24) and short-sighted within-host selection (51).

### Coincidental Evolution

According to the coincidental evolution hypothesis, parasite-mediated morbidity and mortality are what Gould and Lewontin (52) likened to the spandrels of gothic churches. While these structural necessities may frame the frescos and paintings within, that is not the reason for their existence. They are architectural constraints. Analogously, the factors responsible for the virulence of a microparasite in an infected host may have evolved for some purpose other than to provide the parasite an advantage within a host or its transmission to other hosts.

It would be difficult to account for the evolution of botulism toxin by selection favoring *Clostridium botulinum* that kill people who eat improperly canned food. The same argument could be made for the toxins of *C. tetanae* and possibly for those produced by other free-living *Clostridia*. Although these organisms may proliferate in humans, they are soil bacteria, and the effects of the toxin may not contribute to their capacity to colonize, proliferate, and be maintained in humans or to their capacity to be transmitted between human hosts. How many other microparasite-induced symptoms, and the resulting host morbidity and mortality, provide no advantage to that microbe in (or on) a host or its transmission between hosts? Did the lipopolysaccharides and other components of bacterial cell walls and cell membranes evolve because the fitness of bacteria expressing them is enhanced by "endotoxin"—induced overresponse of the immune system responsible for the morbidity and mortality of sepsis (53)? Do the toxins confer an advantage on *E. coli* O157 or *Staphylococcus aureus* (or the plasmids and phages that code for these toxins) because they produce, sometimes lethal, symptoms in infected hosts, hemolytic uremic and toxic shock syndromes, respectively? An earlier paper on this subject (24) argued that the adhesins produced by the *E. coli* responsible for the morbidity of symptomatic urinary tract infections evolved and are maintained to facilitate colonization of the gut. The painful symptoms of urinary tract infections generated by an inflammatory response to these adhesins may confer no advantage for the *E. coli* expressing them in the urinary tract and may in fact lead to the clearance of those bacteria (24).

Each of the symptom-inducing toxins and adhesins described above, as well as many other so-called "virulence determinants" (54) may indeed facilitate the microparasite's ability to colonize, proliferate, or be maintained in infected hosts, and/or be transmitted between hosts. This certainly sounds reasonable for many virulence determinants, e.g., the somatic cell invasiveness mechanisms of *Shigella*, the capsules of *Streptococcus*, the diarrhea-inducing toxins produced by *Vibrio cholerae*, and the sneezing and coughing induced by rhinoviruses. On the other hand, it is necessary to formally test this hypothesis that these symptoms have that effect and reject the alternative, that the morbidity and mortality generated by the expression of a specific virulence determinant provides neither a within- or between-host (infectious transmission) advantage to the parasite.

### Short-Sighted Evolution

Natural selection is a local phenomenon. Characters that confer a survival or replication advantage on the individual organisms that express them at a given time or in a given habitat will be favored and evolve at that time and in that habitat. Whether the expression of those temporally or locally favored characters will increase or reduce the fitness of that organism at other times or in other habitats is irrelevant. Also irrelevant is whether a locally favored character makes the population better or less adapted to its environment at large or augments the likelihood of its survival in the future. This myopia is a fundamental premise of the theory of evolution by natural selection and the basis of the short-sighted evolution hypothesis for microparasite virulence (51).

Within an infected vertebrate host, microparasite populations go through many replication cycles and may achieve very high densities. They may also reside and proliferate in many different subhabitats (tissues and cells) and confront a variety of different and ever-changing constitutive and inducible host defenses which may, sequester, kill, or in other ways inhibit their proliferation. As a consequence of classic mutation, transposition, and recombination, genetic variability will be continually generated in the populations of infecting microbes. Mutant or recombinant microparasites that are better able to 1) avoid being done in or inhibited by the host's defenses; 2) proliferate in the host; or 3) invade and replicate in novel habitats, tissues, and cells where there is less competition from members of its species would have an advantage in that host. This would occur even when the expression of the characters responsible for that local advantage reduces likelihood of the transmission to other hosts. Stated another way, the morbidity or mortality caused by a microparasite infection could be the result of the within-host evolution that is short-sighted because that virulence actually reduces the rate at which that parasite is transmitted to other hosts.

Three examples of microparasite virulence that could be products of this mode of evolution can be considered (51). For two of these examples, bacterial meningitis and poliomyelitis, many human hosts are infected by the responsible microparasites, primarily *Haemophilus influenzae*, *Neisseria meningitidis*, and *Streptococcus pneumoniae* for meningitis and poliovirus for poliomyelitis, but very few manifest the symptoms of these infections. In the case of meningitis, the neurologically debilitating and sometimes fatal symptoms of the infection are a consequence of an inflammatory response against the bacteria entering and proliferating in the cerebral spinal fluid. These meningitis-causing bacteria normally reside in the nasopharyngeal passages and are transmitted by droplet infection. The cerebrospinal fluid is, at least with respect to their infectious transmission, a dead end. On the other hand, bacteria capable of invading and proliferating in that habitat could have a local advantage as there are no other competing populations and only modest defenses. An analogous argument can be put forth for poliovirus. Symptomatic infections with this virus are caused by their invasion of and proliferation in the neurologic tissue of the central nervous system. Poliovirus normally replicates in the mucosal cells of the mouth, throat, and intestines and is transmitted by the oral-fecal route. Poliovirus virions proliferating in the central nervous system would almost certainly not be transmitted. The evidence in support of short-sighted evolution for the virulence of these specific microparasites is mostly circumstantial (51). On the other hand, short-sighted evolution for the virulence of specific microparasites is a hypothesis that can be tested. If the hypothesis is valid, the microparasites responsible for the symptoms would be genetically different from their ancestors that infected the host and better adapted for proliferation in the site of the symptoms than the ancestors themselves.

The third example of short-sighted evolution of virulence considered, HIV, is different from the other two in that virtually every human infected with this retrovirus that does not die of other causes, eventually manifests and succumbs to AIDS. However, although the case mortality of HIV infection may approach unity, as measured by the rate of mortality (deaths per unit of time), from an epidemiologic perspective, HIV is not a very virulent virus. There is substantial variation in the time between infection and the onset of AIDS. On average in industrialized countries, the term of this infection is 8 to 10 years (55). During the early phase of an HIV epidemic, most transmission of the virus occurs during the initial viremia, probably before seroconversion and certainly before the onset of AIDS (37, 56). It is not at all clear how the transmissibility of HIV virions during this early phase of the infection is related to the time of onset of AIDS. HIVs that are more transmissible early in the infection may lead to an earlier onset of AIDS. If this is the case and all else were equal, increasing opportunities for transmission during the epidemic phase would favor increases in HIV virulence (36, 37). However, there may be no association between HIV's capacity to be transmitted early and the time of onset of AIDS, or the time until the onset of AIDS may increase with the transmissibility of the virus during the early phase of the infection. Under either of these conditions, selection during the epidemic phase of the disease would favor more transmissible but less virulent HIVs.

In the course of HIV infection, the HIV population undergoes continuous genetic changes. In fact, in a number of hypotheses of HIV pathogenesis, AIDS is a consequence of mutation and selection in the HIV population that occurs during the course of the infection in individual hosts (57-60), i.e., short-sighted, within-host evolution. Albeit different in their details, all of these hypotheses are consistent with what is known about HIV infection, and all can account for the course of these infections and variable time of onset of AIDS.

### Experimental Evolution Meets Experimental Epidemiology

Results of recent studies by population and evolutionary biologists predict at least three ways by which the virulence of microparasites can be favored and will be maintained by natural selection. 1) Direct selection: there is a positive relationship between the parasite's virulence and its rate of infectious transmission; 2) coincidental evolution: the parasite's virulence is due to character(s) favored and maintained by selection for some other function and the expression of those virulence determinants in an infected host does not confer a net advantage or disadvantage in the parasite population at large; and 3) short-sighted, within-host, evolution: the parasites responsible for the morbidity and mortality of an infection are selected for within the host because of a local advantage, and that evolution reduces the rate at which that locally adapted parasite is transmitted between hosts.

At this time, these predictions are based almost entirely on general theory and retrospective interpretations of epidemiologic and other observations about specific microparasites. Although this theory and these interpretations may be appealing, in a formal Popperian sense (61), almost all the mechanisms postulated for the evolution of virulence of specific microparasites are no more than untested hypotheses. However, unlike most evolutionary hypotheses, those about the evolution of microparasite virulence can be tested and rejected with prospective, experimental studies with laboratory animal and plant hosts. These tests could be at two levels; first, tests of the validity of the assumptions behind these models of the evolution of virulence and second by tests of the predictions made from the consideration and analysis of these models.

For the direct selection hypothesis, it is essential to demonstrate a positive relationship between a microparasite's virulence and its rate of (or capacity for) infectious transmission. For mammalian hosts, protocols exist for determining this relationship (30-32). The object would be to estimate the densities of microbes at the sites of transmission (e.g., feces, nasal passages) during the entire course of the infection. Moreover, it would be useful to separately test the colonization ability and virulence of the microbes from these sites. According to the coincidental evolution hypothesis, it is possible that the virulence determinant responsible for morbidity and mortality in the host provides a local advantage to the parasite expressing it; whether it does or not could be tested with competition experiments between strains of that microparasite that are isogenic save for that virulence determinant. The genetic basis of many virulence determinants are known, and it should be possible to construct these strains. However, unlike in the direct selection hypothesis, in coincidental evolution, microbes expressing the virulence determinants should not be over represented at the sites of infectious transmission. Under the short-sighted evolution hypothesis, microbes isolated from the tissues and organs responsible for the symptoms of the infection (e.g., in the cerebrospinal fluid) should be better adapted for proliferation in those organs and tissues than the originally infecting strain from which they were derived. This could be tested with pairwise competition experiments between the original and potentially evolved strains injected at the site of the symptoms with a common, genetically marked competitor of that parasite. Here, too, it is necessary to demonstrate that the strain responsible for the symptoms is not overrepresented at the site of infectious transmission.

To test the prediction of the direct selection hypothesis and to exclude that mechanism in tests of the coincidental and short-sighted alternatives, it is necessary to study the epidemiology of the microparasites as well as their within-host properties. For bacteriophages and bacteria this is a relatively easy task, e.g., testing Abedon's hypothesis (47) about the direct relationship between the density of sensitive bacteria and selection for latent period length (a measure of virulence) and burst size (a measure of transmissibility). For eukaryotic hosts, this kind of study is going to be more difficult and, at this time, may not be possible. The basic protocols for experimental studies of the epidemiology of bacterial and viral infections of laboratory mice were developed and successfully employed a long time ago (32, 62).[5] However, experiments of these types are costly, labor-intensive, and time-consuming, and because of concerns about animal rights, it may be difficult to get permission to do these experiments with mice or other higher vertebrates. On the other hand, experiments of this type with insects and other invertebrate animal hosts as well as plants would be tenable and valuable as tests of the general theory, albeit less immediately relevant to the evolution and maintenance of virulence in human pathogens.

### Acknowledgments

I thank Marc Lipsitch, Deiter Ebert, Jim Bull, David Thaler, and Tomoko Steen for reading this manuscript and for many insightful comments and suggestions, most of which I agreed with and a few of which I've incorporated. This endeavor was supported by a grant from the National Institutes of Health, GM33782.

*Dr. Levin is professor of biology at Emory University and director of the Graduate Program in Population Biology Ecology and Evolution.*

[5] Greenwood and colleagues (32), studied microparasites with different transmissibilities ("infectivity") and virulence.In one replica of their study of pasteurellosis (due to infections with a bacterium they call *Pasturella muriseptica* in experimental populations of mice, they report "the appearance of a variant that had gained infectivity and retained, or perhaps increased, its original virulence." However, in general, their study and Fenner's (62) provide little information about the direction of natural selection in these microparasite populations. With respect to evolutionary questions, these were wait-and-see experiments. Only one strain of microparasite was introduced into each population, and it was necessary to wait for mutations that changed their virulence or transmissibility. More information about the direction of selection and a better test of these evolutionary hypotheses could be obtained in these types of experiments if two or more genetically marked strains of microparasites with different virulence and transmissibility were introduced simultaneously and were allowed to compete.

*He is a population and evolutionary biologist, who, like a number of others of his ilk, recently discovered infectious disease. Currently he and the postdoctoral fellows and students working with him are doing theoretical (mathematical modeling) and experimental research on the within-host population dynamics of bacterial infections and their treatment, and the epidemiology, population genetics and evolution of antibiotic resistance.*

### References

1. Schrag S, Wiener P. Emerging infectious diseases: what are the relative roles of ecology and evolution? Trends in Ecology and Evolution 1995; 10: 319-23.
2. Wallace B. Can "stepping stones" form stairways? American Naturalist 1989; 133: 578-79.
3. Darwin C. The descent of man and selection in relation to sex. New York: Random House, Modern Library, 1871 (reprinted 1960).
4. Haldane JBS. Disease and evolution. La Ricerca Scientifica 1949; 19:68-76.
5. Garnett GP, Antia R. Population biology of virus-host interactions. In: Morse SS, editor. The evolutionary biology of viruses. New York: Raven Press, 1994:51-73.
6. Ewald PW. The evolution of infectious disease. Oxford, UK: Oxford University Press, 1994.
7. Bull JJ. Virulence. Evolution 1994; 48:1423-37.
8. Frank SA. Models of parasite virulence. Q Rev Biol 1996;71:37-78.
9. Dubos R. Man adapting. New Haven, CT: Yale University Press, 1965.
10. Burnet FM, White DO. Natural history of infectious diseases. Cambridge, UK: Cambridge University Press, 1972.
11. Mims C, Dimmock N, Nash A, Stephen J. Mims' pathogenesis of infectious disease. 4th ed. San Francisco: Academic Press, 1995.
12. May RM, Anderson RM. Parasite host coevolution. In: Futuyama DJ, Slatkin M, editors. Coevolution. Sunderland, MA: Sinauer, 1983:186-206.
13. Essex M, Kanki PJ. The origin of the AIDS virus. Sci Am 1988; 259: 64-100.
14. Leigh Brown AJ. Holmes EC. Evolutionary biology of the human inmunodeficiency virus. Annual Review of Ecology and Systematics 1994; 25:127-62.
15. Davis BD, et al. Microbiology. 4th ed. Philadelphia: Lippincott, 1990.
16. Waters AP. Higgins DG, McCutchan TF. *Plasmodium falciparum* appears to have arisen as a result of lateral transfer between avian and human hosts. Proc Natl Acad Sci USA 1991; 88: 3140-4.
17. Allison MR, Mendoza O, Pezziam A. Documentation of a case of tuberculosis in pre-Columbian America. Am Rev Resp Dis 1973; 107: 985.
18. Bates JH, Stead WW. The history of tuberculosis as a global epidemic. Med Clin North Am 1993; 77: 1205-17.
19. Allison AC. Protection afforded by sickle cell trait against malarial infection. Br Med J 1954; 2:290-4.
20. Luzzatto L, Usanga EA, Shunmugam R. Glucose 6-phosphate dehydrogenase deficient red cells: resistance to infection with malarial parasites. Science 1969; 164: 839-41.
21. Miller LH, Mason SJ, David FC, McGinnis MH. The resistance factor to *Plasmodium vivax* in Blacks. N Engl J Med 1976; 295:302-4.
22. Hill AVS, Allsopp CEM, Kwaitkowski D, Ansty NM, Twumasi P, Rowe PA, et al. Common West African HLA antigens are associated with protection from severe malaria. Nature 1991; 252:595-600.
23. Lurie MB. Resistance to tuberculosis: experimental studies of native and acquired defensive mechanisms. Cambridge, MA: Harvard University Press, 1964.
24. Levin BR, Svanborg-Eden C. Selection and the evolution of virulence in bacteria: an ecumenical excursion and modest suggestion. Parasitology 1990; 100:S103-15.
25. Anderson RA, May RM. Infectious diseases of humans: dynamics and control. Oxford, UK; Oxford University Press, 1991: vii, 757.
26. Anderson RM, May RM. Co evolution of hosts and parasites. Parasitology 1982; 85:411-26.
27. Levin BR, Alison AC, Bremermann HJ, Cavali-Storza LL, Clarke BC, Frentzel-Beymem R, et al. Evolution of parasites and hosts (group report). In: Anderson RM, May RM, editors. Population biology of infectious diseases. Berlin: Springer, 1982:212-43.
28. Fenner F, Cairns J. Variation in virulence in relation to adaptation to new hosts. In: Burnet FM, Stanley WM, editors. The viruses: biochemical biological and biophysical properties. New York: Academic Press, 1959:225-49.
29. Fenner F, Ratcliffe FN. Myxomatosis. Cambridge, UK: Cambridge University Press, 1965.
30. Fenner FM, Day MF, Woodroofe GM. Epidemiological consequences of the mechanical transmission of myxoma by mosquitoes. Journal of Hygiene 1956; 54:284-303.
31. Mead-Briggs AR, Vaughan JA. The differential transmissibility of myxoma virus strains of differing virulence grades by the rabbit flea *Spilopsyllus cuniculi* (Dale). Journal of Hygiene 1975; 75:237-47.
32. Greenwood M, Hill AB, Topley WWC, Wilson J. Experimental epidemiology. London: Medical Research Council, 1936:209:1-204.
33. Herre EA. Population structure and the evolution of virulence in nematode parasites in fig wasps. Science 1993; 259:1442-5.
34. Ebert D. Virulence and local adaptation of a horizontally transmitted parasite. Science 1994; 265:1084-6.
35. Ewald PW. Host parasite relations, vectors, and the evolution of disease severity. Annual Review of Ecology and Systematics 1983; 14:465-85.
36. Lipsitch M, Nowak ML. The evolution of virulence in sexually transmitted HIV/AIDS. J Theor Biol 1995; 174:427-40.
37. Levin BR, Bull JJ, Stewart FM. The intrinsic rate of increase in HIV/AIDS: epidemiological and evolutionary implications. Math Biosci 1996; 132:69-96.
38. Levin BR, Lenski RE. Coevolution of bacteria and their viruses and plasmids. In: Futuyama DJ, Slatkin M, editors. Coevolution. Sunderland, MA: Sinauer Associates, 1983:99-127.

39. Bull JJ, Molineux IJ, Rice WR. Selection of benevolence in a host parasite system. Evolution 1991; 45:875-82.
40. Sasaki A, Iwasa Y. Optimal growth schedule of pathogens within a host: switching between lytic and latent cycles. Theor Popul Biol 1991; 39:201-39.
41. Antia R, Levin BR, May RM. Within-host population dynamics and the evolution and maintenance of microparasite virulence. American Naturalist 1994; 144:457-72.
42. Bonhoeffer SA, Nowak MA. Mutation and the evolution of virulence. Proc R Soc Lond B Biol Sci 1994; 258:133-40.
43. Nowak MA, May RM. Superinfection and the evolution of parasite virulence. Proc R Soc Lond B Biol Sci 1994; 255:81-5
44. Koella JC, Antia RN. Optimal pattern of replication and transmission for parasites with two stages in their life cycle. Theor Popul Biol 1995; 41:277-91.
45. Bonhoeffer S, Nowak MA. Intra-host versus inter-host selection: viral strategies of immune function impairment. Proc Nat Acad Sci USA 1994; 91:8062-6.
46. Lenski RE, May RM. The evolution of virulence in parasites and pathogens: reconciliation between two competing hypotheses. J Theor Biol 1994; 169:253-65.
47. Abedon ST. Selection for bacteriophage latent period length by bacterial density: a theoretical examination. Microbial Ecology 1989; 18:79-88.
48. Stewart FM, Levin BR. The population biology of bacterial viruses: why be temperate? Theor Popul Biol 1984; 26:93-117.
49. Lipsitch M., et al. The population dynamics of vertical and horizontally transmitted parasites. Proc R Soc Lond B Biol Sci 1995; 260:321-7.
50. Lipsitch M, Siller S, Nowak MA. The evolution of virulence in pathogens with vertical and horizontal transmission. Evolution 1996 (in press).
51. Levin BR, Bull JJ. Short-sighted evolution and the virulence of pathogenic microorganisms. Trends Microbiol 1994; 2:76-81.
52. Gould SJ, Lewontin RC. The spandrels of San Marco and the pangalossian paradigm: a critique of the adaptationist programme. Proc R Soc Lond B Biol Sci 1979; 205:581-98.
53. Whitnack E. Sepsis. In: Schaechter M, Medhoff G, Eisenstein BI, editors. Mechanisms of microbial disease. Baltimore: Williams & Wilkins, 1993: 770-8.
54. Finlay BB, Falkow S. Common themes in microbial pathogenicity. Microbiol Rev 1989; 52:210-30.
55. Fauci AS. Multifactorial nature of human immunodeficiency virus disease: implications for therapy. Science 1993; 262:1008-11.
56. Jacques A, Koopman JS, Simon CP, Longini IM. The role of primary infections in epidemics of HIV infections in gay cohorts. J Acquir Immune Defic Syndr 1994; 7:1169-84.
57. Nowak MA, Anderson RM, McLean AR, Wolfs TFW, Goudsmit J, May RM. Antigenic diversity thresholds and the development of AIDS. Science 1991; 254:963-9.
58. McLean AR. The balance of power between HIV and the immune system. Trends Microbiol 1993; 1:9-13.
59. Mittler JM, Antia R, Levin BR. Population dynamics of HIV pathogenesis. Trends in Ecology and Evolution 1995; 10:224-7.
60. Mittler JM, Levin BR, Antia R. T-cell homeostasis, competition and drift: AIDS as HIV-accelerated senescence of the immune repertoire. J Acquir Immune Defic Syndr Hum Retrovirol (in press).
61. Popper KR. The logic of scientific discovery. New York: Harper, 1965: 479.
62. Fenner F. The epizootic behaviour of mousepox (infectious ectromelia of mice) II. The course of events in long-continued epidemics. J Hygiene 1948; 46:383-93.
